# Growth Response and Recovery of *Corynebacterium glutamicum* Colonies on Single-Cell Level Upon Defined pH Stress Pulses

**DOI:** 10.3389/fmicb.2021.711893

**Published:** 2021-10-01

**Authors:** Sarah Täuber, Luisa Blöbaum, Volker F. Wendisch, Alexander Grünberger

**Affiliations:** ^1^Multiscale Bioengineering, Technical Faculty, Bielefeld University, Bielefeld, Germany; ^2^CeBiTec, Bielefeld University, Bielefeld, Germany; ^3^Genetics of Prokaryotes, Faculty of Biology, Bielefeld, Germany

**Keywords:** microfluidics, single-cell cultivation, *C. glutamicum*, pH stress pulses, pH homeostasis

## Abstract

Bacteria respond to pH changes in their environment and use pH homeostasis to keep the intracellular pH as constant as possible and within a small range. A change in intracellular pH influences enzyme activity, protein stability, trace element solubilities and proton motive force. Here, the species *Corynebacterium glutamicum* was chosen as a neutralophilic and moderately alkali-tolerant bacterium capable of maintaining an internal pH of 7.5 ± 0.5 in environments with external pH values ranging between 5.5 and 9. In recent years, the phenotypic response of *C. glutamicum* to pH changes has been systematically investigated at the bulk population level. A detailed understanding of the *C. glutamicum* cell response to defined short-term pH perturbations/pulses is missing. In this study, dynamic microfluidic single-cell cultivation (dMSCC) was applied to analyze the physiological growth response of *C. glutamicum* to precise pH stress pulses at the single-cell level. Analysis by dMSCC of the growth behavior of colonies exposed to single pH stress pulses (pH = 4, 5, 10, 11) revealed a decrease in viability with increasing stress duration *w*. Colony regrowth was possible for all tested pH values after increasing lag phases for which stress durations *w* were increased from 5 min to 9 h. Furthermore, single-cell analyses revealed heterogeneous regrowth of cells after pH stress, which can be categorized into three physiological states. Cells in the first physiological state continued to grow without interruption after pH stress pulse. Cells in the second physiological state rested for several hours after pH stress pulse before they started to grow again after this lag phase, and cells in the third physiological state did not divide after the pH stress pulse. This study provides the first insights into single-cell responses to acidic and alkaline pH stress by *C. glutamicum*.

## Introduction

Various environmental fluctuations influence the growth and physiology of bacteria (Lara et al., 2006). One decisive parameter is the pH value, which impacts the solubilities of nutrients and cellular metabolism (Follmann et al., 2009b; Krulwich et al., 2011; Rosenthal et al., 2017). External pH fluctuations have important impacts on the intracellular pHs of bacteria. A change in the intracellular pH affects enzyme activity, protein stability, solubilities of trace elements and proton motive force (PMF) (Olson, 1993; Follmann et al., 2009b; Krulwich et al., 2011; Rosenthal et al., 2017). The intracellular pH value is maintained by pH homeostasis (Haynes et al., 2019). The maintenance of pH homeostasis in bacteria includes a wide range of constitutive and regulated mechanisms, which have been investigated most extensively in *Escherichia coli* (Slonczewski et al., 1981; Foster, 2004). During acid stress, the presence of potassium and other osmolytes in the medium is essential to maintaining the cytoplasmic pH (Martinez et al., 2012). *E. coli* has three different mechanisms for resistance to acidic pH values, one glucose catabolite-repressed system and two amino acid decarboxylase-dependent systems (Tucker et al., 2002). Sodium proton antiporters such as MDfA and NhaA lead to resistance to alkaline pHs (Lewinson et al., 2004). This work is focused on an industrially relevant workhorse, the Gram-positive bacterium *Corynebacterium glutamicum*. Historically, it has been used for production of amino acids such as L-glutamic acid, L-lysine and L-threonine (Wendisch, 2020). *C. glutamicum* exhibits many beneficial attributes as an industrial host, such as fast growth, cultivation to high cell densities, genetic stability, and a broad spectrum of possible carbon sources (Lee et al., 2016). *C. glutamicum* is a neutralophilic organism that can maintain an internal pH of 7.5 ± 0.5 in spite of environmental fluctuations between pHs of 5.5 and 9.0 (Follmann et al., 2009b). Outside this range, the internal pH collapses, and finally, pH homeostasis fails. In acidic environments, a significant amount of reactive oxygen species (ROS) is produced, which leads to oxidation of methionine and cysteine residues of proteins or iron sulfur clusters as well as to DNA damage (Follmann et al., 2009b). As a result, the metabolism may change, e.g., the iron starvation response is activated, consequently affecting the TCA cycle and NAD and methionine syntheses. As a result of reduced methionine synthesis, cysteine accumulates, which is toxic in acidic environments (Follmann et al., 2009b). Another important mechanism for pH homeostasis in acidic environments is potassium uptake via potassium channels (Kitko et al., 2010; Ochrombel et al., 2011). This stabilizes the PMF, which is essential for *C. glutamicum* growth in acidic and alkaline environments. This electrochemical proton gradient across the bacterial cell membrane is kept constant by ion transporters. In acidic environments, the gradient increases so that the electrochemical potential is adjusted by potassium flux (Follmann et al., 2009a). In addition to inorganic acidic environments, pH shifts can also be induced by organic acids, which affect not only the H^+^ concentration but also the available carbon source (Jakob et al., 2007). In alkaline environments, much less is known about the molecular adaption mechanisms of *C. glutamicum*. An MdfA homolog is missing, and the possible involvement of further sodium proton antiporters in the pH reaction is not clear. Genes coding for homologous proteins of amino acid decarboxylase, e.g., AdiCA, GadABC and CadAB, are also absent (Kalinowski et al., 2003; Follmann et al., 2009b). The generation of a considerable electrochemical potential across the cell membrane is critical for the entry of protons, typically via cation/proton antiporters (Krulwich et al., 2011). Until now, Mrp-Type Na^+^(Li^+^)/H^+^ antiporters 1 and 2 were found to contribute to resistance in alkaline environments (Xu et al., 2018). It was also postulated that the concentrations of proteins of the succinate dehydrogenase complex and F_0_F_1_-ATP synthesis are increased (Barriuso-Iglesias et al., 2008). The reader is referred to Guo et al. (2019) for a detailed summary of the known mechanism and suggested strategies with which *C. glutamicum* may cope with pH stress.

The current state of knowledge related to the *C. glutamicum* response to pH stress is based on bulk population studies with microbial cells cultivated in small bioreactors or shaking flasks (Jakob et al., 2007; Follmann et al., 2009b). Using these methods, representative information cannot be gathered for individual cells (Lindström and Andersson-Svahn, 2010), and cell-to-cell heterogeneity remains unclear (Lindemann et al., 2019). Furthermore, traditional cultivation lacks temporal precision and spatial resolution, e.g., due to slow mixing in large volumes to perform stress response experiments, such as those with defined stress pulses, or even the investigation of oscillating stress conditions (Lara et al., 2006). However, what has been lacking is information on how cells respond to pH stress at the individual level.

Microfluidic methods offer the opportunity to investigate microbial behavior at the single-cell level. Here, microfluidic single-cell cultivation and analysis systems allow the cultivation of bacteria under defined environmental conditions and offer the analysis of cellular behavior with high spatial and temporal resolution through live-cell imaging (Grünberger et al., 2014; Dusny et al., 2015). Depending on the microfluidic systems, hundreds of cells can be cultured and analyzed in a high-throughput manner. In the last 20 years, microfluidic single-cell cultivation systems have been successfully applied to investigate the cellular heterogeneity of different physiological phenomena such as the cell cycle (Elowitz and Leibler, 2000), aging (Wang et al., 2010), growth (Wang et al., 2010; Unthan et al., 2014; Binder et al., 2016), etc. Recently, the first dynamic microfluidic single-cell cultivation (dMSCC) systems for cultivation and analyses of cells during defined stress pulses and oscillations was reported for *C. glutamicum* (Täuber et al., 2020). The dMSCC setup was used to analyze different oscillation frequencies that show the influence of nutrient limitations on *C. glutamicum*. This dMSCC system was used here because it supports fast and precise medium changes, and its high degree of parallelization provides sufficient data for rigorous statistical evaluation and, for the first time, allows control measurements of oscillation parameters to be recorded in parallel with data measurements.

In this work, the influences of different pH values and pH pulses on the growth of *C. glutamicum* were systematically investigated. The dMSCC system allowed us to investigate the behavior of populations at the single-cell level and provided single-cell data for responses to pH stress pulses, which had not been analyzed before. First, the growth of *C. glutamicum* at different constant pH values between 5 and 10 was investigated at the colony level. In addition, responses at the colony level and single-cell level after different stress pulses of pH 4, 5, 10 and 11 with stress durations *w* between 5 min and 9 h were investigated to determine how many cells were viable after the stress and to identify the regrowth behaviors resulting after application of the different stress conditions. Furthermore, the recovery behavior of single cells after different stress durations *w* was investigated. The heterogeneity of single cells was investigated to determine whether different subpopulations could be identified that differ in their adaptive responses to pH.

## Materials and Methods

### Precultivation, Bacterial Strain, Medium

In this study, the bacterial strain *C. glutamicum* WT ATCC 13032 was cultivated in CGXII media at 30°C (Unthan et al., 2014). Each medium component was autoclaved. Afterward, the pH value of the medium was adjusted with either HCl, KOH, H_3_PO_4_ or NaOH. Prior to dMSCC, the medium was sterile filtered. For all microfluidic experiments, CGXII medium without MOPS was used.

Overnight precultures of *C. glutamicum* were inoculated from glycerol stock and then grown in 10 ml of CGXII medium (with MOPS) in 100 mL baffled flasks on a rotary shaker operating at 120 rpm. Cells from the overnight culture were transferred to inoculate the main culture with a starting OD_600_ of ~0.05. When the culture exhibited an OD_600_ of ~0.2, the cells were seeded in the microfluidic device. After seeding, the cells were dynamically perfused with CGXII medium at pH 7 and CGXII medium with varying pH values according to the protocol described below.

### Live/Dead Staining

Medium with a final propidium iodide concentration of 1 μM (stock solution: 1 mM in water) (Krämer et al., 2016) was used for dead staining (propidium iodide; Cayman Chemical Company, USA). The pH was then adjusted, and the medium was sterile filtered to prevent channel blockage. dMSCC with individual stress pulses of pH 5 for 2, 6 and 9 h and pH 10 for 2 and 6 h were performed. For a more detailed description, see “Setup and Microfluidic Cultivation.”

### Chip Preparation

For the PDMS soft lithography mold, a silicon wafer was fabricated. The detailed processing steps for wafer fabrication are shown in Täuber et al. (2020).

The wafer was covered with PDMS at a ratio of 10:1 (Sylgard 184 Silicone Elastomer, Dow Corning Corporation, USA). Afterward, the wafer was degassed in an exicator for 20 min and backed for 2 h at 80°C (Universal cupboard, Memmert GmbH, Germany). After this, the PDMS chip was cut from the wafer, inlets and outlets were punched with a 0.75 mm biopsy puncher (Reusable Biopsy Punch, 0.75 mm, WPI, USA) and cleaned with isopropanol three times. The cover glass (D 263 T eco, 39.5 ×34.5 ×0.175 mm, Schott, Germany) was cleaned just as the PDMS chip. The PDMS chip and the cover glass were O_2_ plasma (Femto Plasma Cleaner, Diener Electronics, Ebhausen, Germany) oxygenized for 24 s and assembled. Afterward, a 2-min post bake at 80°C was performed to strengthen the bonding.

### Chip Design

The chip design published in Täuber et al. (2020) was used with two inlets for the different pH values. Between the inlets, there were several arrays of monolayer cultivation chambers (80 ×90 μm ×750 nm), whereby the different oscillation zones were separated from each other by a channel with a width of 400 μm; thus, the flow profile is much more stable, and five different stress conditions can be tested. The supply channels have a height of ~10 μm and a width of 100 μm.

### Setup and Microfluidic Cultivation

Culture preparation is described in the section “Precultivation, Bacterial Strain, Medium” provided above. For the loading process, the cell suspension from the main culture was inoculated at an OD_600_ of ~0.2 in the microfluidic device. In a randomized process, ~75% of the cultivation chambers were loaded with ~1–4 cells (Probst et al., 2015). When a sufficient number of chambers was filled with single cells, the flow of the cell suspension was stopped, and the flow of medium was started. The medium flow was executed with high precision pressure pumps (Line-up series, Fluigent, Jena, Germany) with pressures for the two-inlet chip of 180 and 20 mbar to start the single stress pulse experiments (for detailed flow profiles, see Supplementary Figure S1). The pulse profiles were applied using a tailor-made cultivation profile implemented in an automated software tool (microfluidic automation tool (MAT), Fluigent, Jena, Germany).

**Figure 1:**
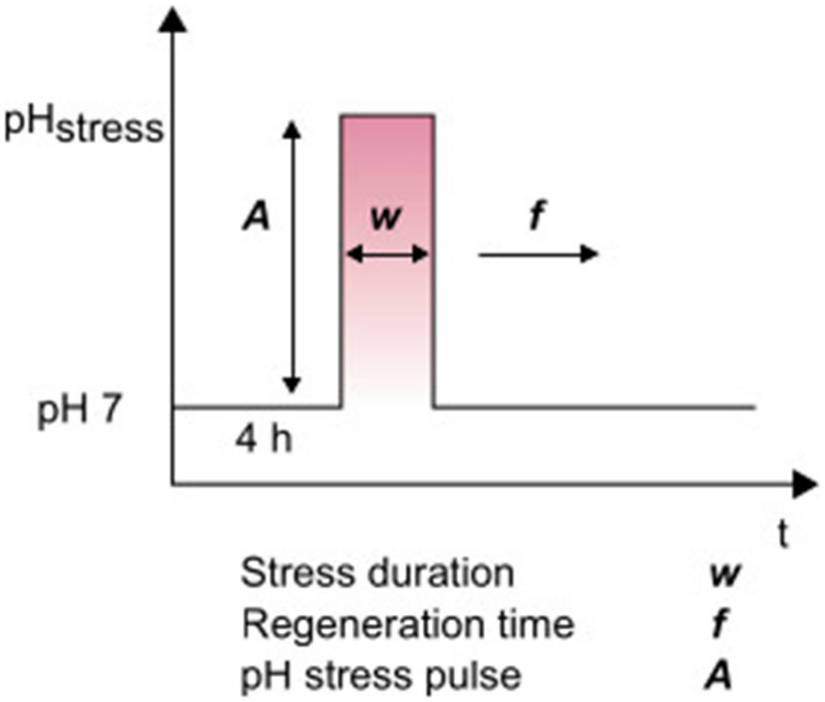
Overview of parameters for dynamically controlled pH values.

In this study, the cells were first adapted for 4 h in a microfluidic device at pH 7 before pH stress was induced. The pH stress pulses (*A*) were varied among pHs of 4, 5, 10 and 11 for different experiments. Single stress pulses were performed with stress durations (*w*) varying between 5 min and 9 h (Figure 1). Afterward, a regeneration time (*f* ) was established until the end of cultivation, usually between 20 and 48 h, to analyze the regrowth of cells. During measurements of the alkaline stress pulses, crystallization sometimes occurred at the boundary layer between the reference medium at pH 7 and the alkaline solutions with pH values of 10 and 11. Magnesium sulfate or magnesium hydroxide probably crystallized out at this boundary layer. The solubility of magnesium hydroxide is 12.5 mg/L (Dean, 1998), while the CGXII medium contains ~10 times this concentration. The data shown here were obtained in the absence of crystals. The analysis of the experiment was stopped when crystallization occurred and the flow profile was significantly altered to guarantee proper interpretation of the data.

### Live-Cell Imaging

Live-cell microscopy was conducted using an inverted automated microscope (Nikon Eclipse Ti2, Nikon, Germany). The microscope was placed in a cage incubator for optimal temperature control at 30°C (Cage incubator, OKO Touch, Okolab S.R.L., Italy). The microfluidic chip was attached to a holder manufactured in-house. The setup was equipped with a 100 × oil objective (CFI P-Apo DM Lambda 100 × Oil, Nikon GmbH, Germany), a DS-Qi2 camera, and an automatic focusing system (Nikon PFS, Nikon GmbH, Germany) to prevent thermal drift during cultivation. One hundred cultivation chambers were manually selected in each experiment by using NIS Elements software (Nikon NIS Elements AR software package, Nikon GmbH, Germany). Images were taken every 10 min with an exposure time of 50 ms.

### Image and Data Analyses

Analyses of image sequences were performed with the open source software Fiji (Schindelin et al., 2012). In the phase contrast images, the cells were separated from the background for each time point with k-mean clustering for background correction. The cluster with the cells was maintained, and the background and intermediate areas between cells were deleted. Cells that were located too close together could not be separated by clustering. Therefore, a watershed transformation was performed. The integrated analysis particle was then used to determine the cell count and cell area for each time point.

**Figure 2:**
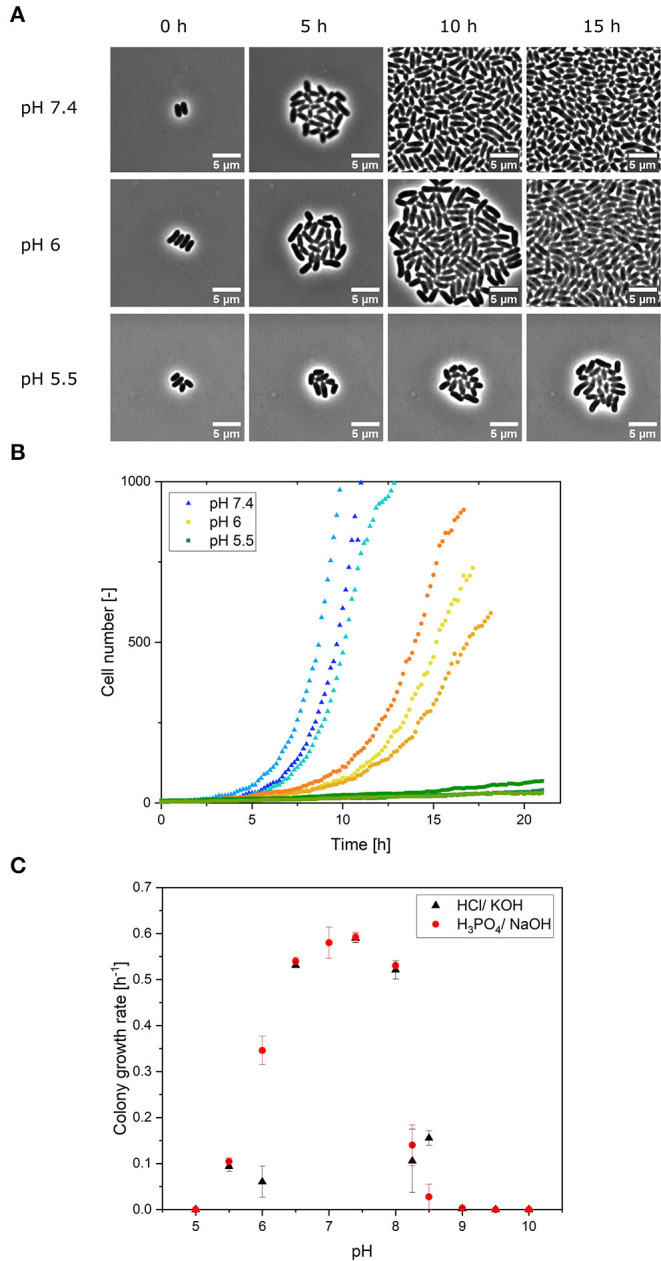
**(A)** Microscope images of growing colonies in constant pH conditions of pH 7.4, 6 and 5.5 at different points in the cultivation time. Scale was set to 5 μm. **(B)** Colony growth of *C. glutamicum* at different, constant pH values (5.5, 6, 7.4) using NaOH and H_3_PO_4_ for pH adjustment. Three colonies (biological replicates) are shown for each pH value in different shades of the same color. **(C)** Optimal pH curve for the colony growth of *C. glutamicum* on a microfluidic chip in CGXII medium. The error bars represent the standard deviation for each pH value. For each pH value experiment, *n* = 3 cultivation chambers were analyzed. Each calculated colony growth rate represents an average of ~512 division events.

**Figure 3:**
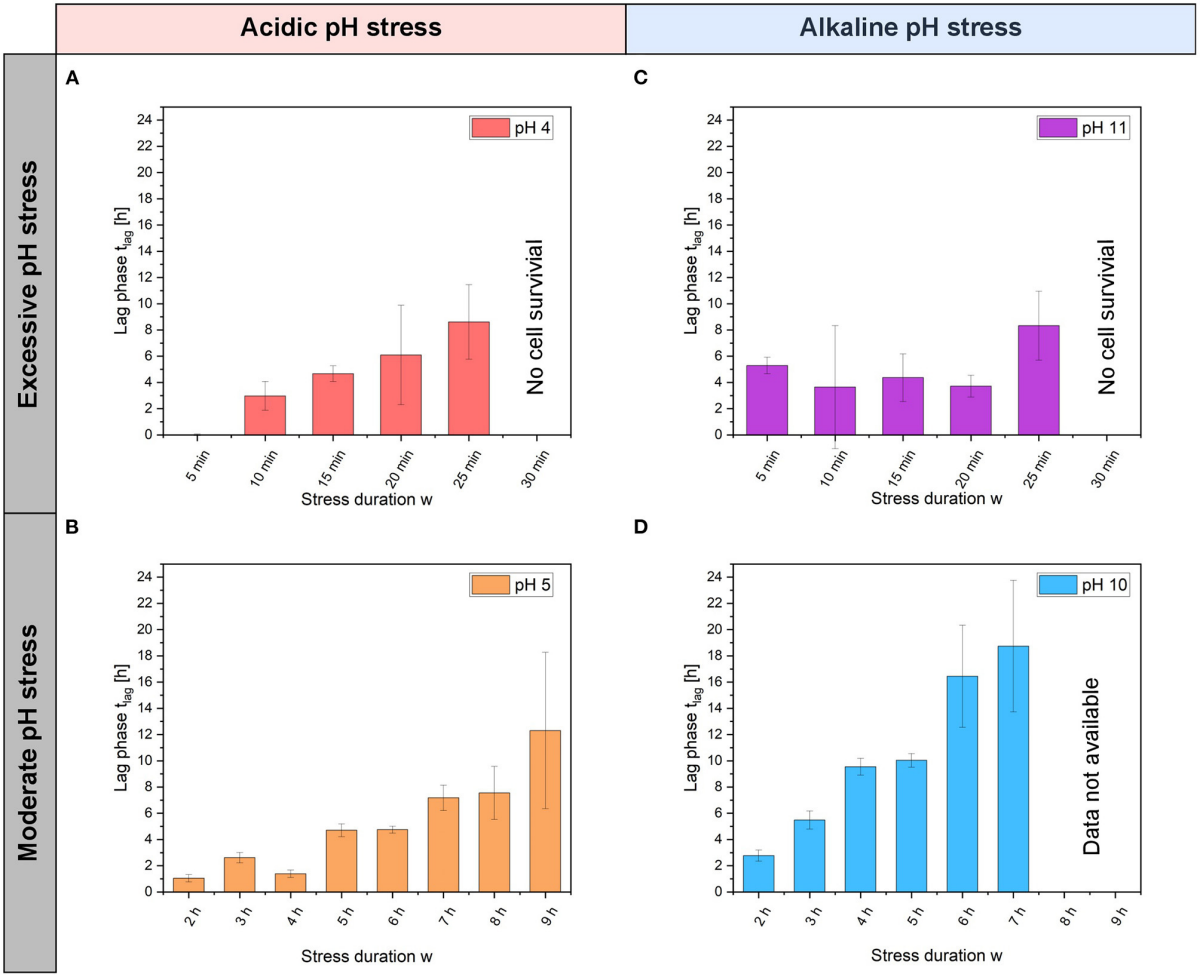
Lag phase of *C. glutamicum* at different pH stress pulses and stress durations *w*. Lag phase *t*_lag_ after acidic **(A)** pH 4 and **(B)** pH 5 stress pulses and after alkaline, **(C)** pH 11 and **(D)** pH 10 stress pulses. Here, error bars represent the standard deviation for each stress duration *w* and each pH value. For each pH experiments with different stress duration *w, n* = 3 cultivation chambers were analyzed.

**Figure 4:**
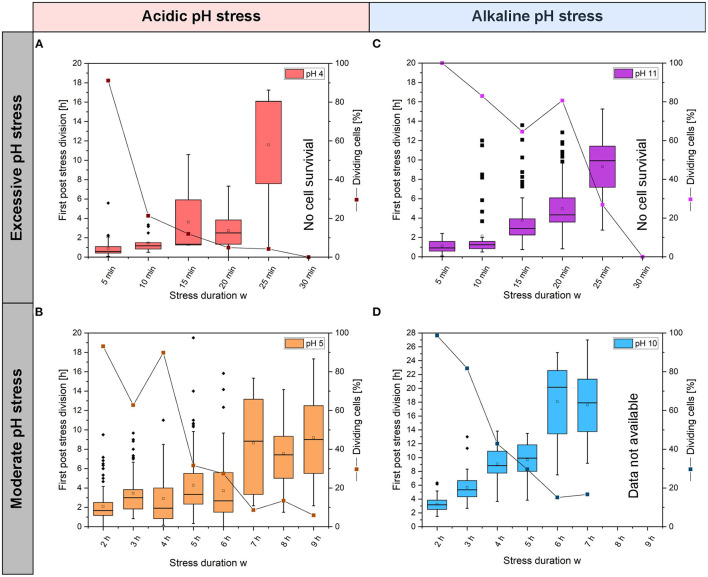
Distributions for the times of first post-stress divisions of single cells and viabilities after acidic **(A)** pH 4 and **(B)** pH 5 stress pulses and after alkaline; **(C)** pH 11 and **(D)** pH 10 stress pulses. The boxplots show the distribution of the first post-stress division, where the longitudinal bar within the box indicates the median of the first post-stress division. The deviation, also called whisker, shows the upper and lower 25% of the first-post-stress division. In addition, outliers are marked with a dot. On average, 97 single cells (min = 38, max = 181) were counted for each pH and stress duration *w* (see Supplementary Table S1).

**Figure 5:**
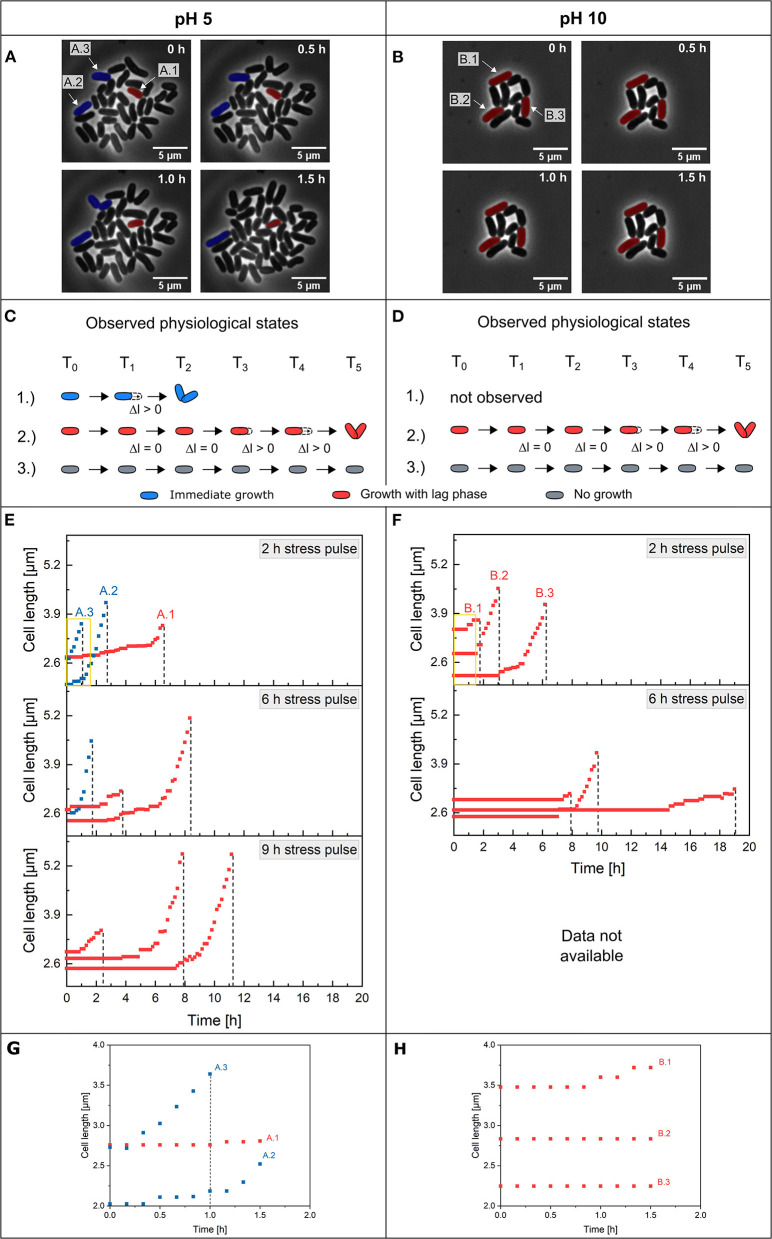
Single-cell adaptation of *C. glutamicum* after pH stress pulses. Microscope images of growing colonies after a 2 h stress pulse of **(A)** pH 5 and **(B)** pH 10 at different post-stress time periods. Cells marked blue could be preadapted cells (A.2 and A.3), and red marked cells are non-adapted cells showing a lag phase before elongation and division (A.1 and B.1–B.3). The scale was set to 5 μm. The observed physiological states regarding regrowth are shown in **(C)** for pH 5 and **(D)** for pH 10. **(E,F)** Cell elongations of single *C. glutamicum* cells occurring after different stress pulses (2, 6 and 9 h) for pH 5 and 10 stress until the first post-stress division. The dashed line marks the first post-stress division of the cells. The data for the 9 h stress pulse of pH 10 was not available due to crystal formation at the boundary layer between the reference medium at pH 7 and the alkaline solutions at pH 10 during dMSCC. **(G,H)** Zoomed-out views of the yellow boxes in **(C,D)** during the first 1.5 h after a 2 h stress pulse. Growth behavior is shown until the time of the first post-division. On average, 113 single cells (min = 77, max = 152) were counted for each pH and stress duration *w*.

Using OriginPro 2019b (OriginLab Corporation, Northampton, USA), the growth curves were plotted, and the colony growth rate was determined by a linear fit to a semilogarithmic plot of the cell number. The mean values of the growth rates were determined and standard deviation to classify the significance were calculated and presented. For each pH value experiment (see Figure 2C) and pH experiments with different stress duration *w* (see Figure 3), *n* = 3 cultivation chambers were analyzed. Dynamic single-cell data illustrated in Figures 4, 5 were analyzed manually. On average, 97 single cells (min = 38, max = 181) were counted for each pH and stress duration *w* for Figure 4, and 113 single cells (min = 77, max = 152) were counted for Figure 5.

The statistical analyses were performed using the function two sample independent *t*-test contained in OriginPro 2019b (OriginLab Corporation, Northampton, USA). Here, the value of t represents the difference relative to the scatter in the sample data and p represents the significance.

## Results

### *Corynebacterium glutamicum* Growth in Constant pH Environments

In a microscopy setup, the dMSCC approach allows us to monitor single cells growing into colonies of up to 1,000 cells in cultivation chambers with a supply of cultivation medium. Colony growth is defined here by the increases in cell numbers in a cultivation chamber that typically starts with one or a few single cells at the beginning of cultivation. In the first set of experiments, microbial colony growth was investigated by dMSCC at different but constant pH values ranging from 5 to 10. Colony growth was monitored by live-cell imaging (Figure 2A), and pH-specific growth rates for each pH were determined from cell numbers (Figure 2B—pH 5.5, 6 and 7.4). Two sets of experiments were performed: the first used HCl and KOH for pH adjustment, and the second used NaOH and H_3_PO_4_. Based on the growth rates, a pH optimum curve for *C. glutamicum* on CGXII was derived with acid or base used for pH adjustment (Figure 2C).

Optimal growth rates were obtained between pH 7 and 7.4 with a maximum growth rate of μ_max_ = 0.59 ± 0.01 h^−1^ at pH 7.4. Exponential growth was observed for all colonies at pH values between pH 6 and 8. Colonies cultivated at pHs between 5 and 6 and between 8 and 9 grew very slowly, so no exponential growth was observed. Linear growth of the cell area was monitored. Consequently, at limiting pH values of 5.5, 8.25 and 8.5, growth was significantly hampered by the pH conditions, and only a small fraction of cells showed division and growth. In constant cultivation with pH values ≤ 5 and ≥ 9, no growth was monitored after 24 h of cultivation. Based on these results, cultivation at pH 4 was not performed, since it is also to be expected that the cells will not grow here, since no growth was already observed at pH 5. The choice of acid or base used to control the medium pH did not influence the colony growth rate in the pH range 6.5–8. However, at very acidic pH values of 5.5 and 6, growth rates were reduced by 2.7-fold [pH 6: *t*_(4)_ = 8.62, *p* = 1.8 ×10^−4^] when the pH of CGXII medium was adjusted with hydrochloric acid instead of phosphoric acid (Figure 2C). There was a significant difference [*t*_(4)_ = 8.62, *p* = 9.9 ×10^−4^] in the average growth rates when the medium was adjusted to pH 5.5 with phosphoric acid (μ_mean_ = 0.105 ± 0.008 h^−1^) and with hydrochloric acid (μ_mean_= 0.029 ± 0.013 h^−1^). A similar effect was observed at an alkaline pH of 8.5; cells in the NaOH-adjusted medium grew significantly slower by 0.85-fold [*t*_(4)_ = −7.09, *p* = 0.002] than cells in the KOH-adjusted medium (Figure 2C). H_3_PO_4_ and NaOH were chosen for the next experiments, as both are also used in the bioreactor (Seletzky et al., 2007).

### *Corynebacterium glutamicum* Growth in Single pH Stress Pulse Experiments

After studying the growth of *C. glutamicum* at constant pH values, the response to a single pH stress pulse was studied. Therefore, pH stress amplitudes *A* between 4 and 11 and various stress durations *w* were tested when a single pH stress pulse was applied after 4 h of growth in dMSCC at pH 7. The aim was to answer the key question of how *C. glutamicum* responds to abrupt pH changes with different amplitudes *A* and stress durations *w*. Therefore, it was determined whether cells continued to grow or stopped growing during the stress pulse and, if so, when regrowth after the pH stress pulse occurred (lag phase). The colony lag phase is defined here as the time between the end of the pH stress pulse and the onset of regrowth of a colony. Dividing cells were defined as the proportion of cells showing regrowth and cell division after the stress pulses gave rise to non-dividing cells. Furthermore, the time at which the first post-stress cell division (first division after the stress pulse represents the individual lag phase) occurred was determined.

### *Corynebacterium glutamicum* Growth at the Colony Level After Various pH Stress Pulses

In the first set of experiments, colony growth was determined after pH stress pulses lasting between 5 min and 9 h. During the pH stress pulse, the colony area neither decreased nor increased (Supplementary Figure S2). After the pH stress pulse, an increase in colony area was observed. The lag phase after pH stress pulses with amplitudes *A* ranging from 4 to 11 are shown in Figure 3. Colonies showed regrowth after stress pulses of pH 5 and 10 with durations longer than 6 h (Figures 3B,D). The lag phase after pH 5 stress pulses increased with increasing stress duration *w*. With stress durations *w* between 2 h and 4 h, the lag phase was only ~2 h, after 9 h of stress the lag phase increased to ~12 h. With pH 10 stress pulses, the lag phase increased from ~2.8 to ~18 h with increasing stress duration *w*. Due to precipitation or crystallization at alkaline pH values, the values for pH 10 stress durations *w* of 8 and 9 h could not be measured (see Materials and Methods section). At pH 4, the cells continued to grow without a lag phase after 5 min of stress.

### *Corynebacterium glutamicum* Growth at the Single-Cell Level After Various pH Stress Pulses

To gain insight into whether *C. glutamicum* shows a homogenous response to pH stress pulses or whether heterogeneity and distinct subpopulations arise, single-cell behavior was analyzed after pH stress pulses by measuring the time until the first post-stress division event (= individual lag phase) after different stress durations *w* and different pH stress amplitudes *A*.

With acidic pH values, the times for first post-stress division showed significant variation (Figures 4A,B). At pH 5, some cells divided directly after the stress phase (stress durations *w* between 2 and 6 h), while other cells in the same colony only divided after a lag phase. After 2 h of stress duration *w*, a normal distribution was observed for the time of the first post-stress division, as expected for a homogeneous population (see Supplementary Figure S4A). The longer the duration of the stress, the larger the median time of the first post-stress division and the broader the time distribution. After a 6 h stress duration *w*, a normal distribution was not observed, and the distribution was wider, indicating the presence of two subpopulations (see Supplementary Figure S4B). Some of the dividing cell populations decreased drastically to ~30% after 4 h, and after 9 h of stress, the viability was only ~6%. Thus, relationships between stress duration *w* and elongation of the lag phase or loss of viability were found (Supplementary Figure S3). At pH 4, the time of the first post-stress division increased with increasing stress duration *w*; after short stress durations *w*, single cells divided in <2 h. After a 25 min stress duration *w*, only a few cells survived (Supplementary Table S1), and the times for the first post-stress division exhibited large variations between 1 and 17 h.

With alkaline stress pulses at pH 11, great variations in the times of single cell post-stress division were observed, which ranged from ~1 h up to ~12 h. After 30 min at pH 11, no cells survived (*n* = 95) (Figure 4C and Supplementary Table S1). At pH 10, stress pulses revealed that the first division after a stress pulse required between ~3 and ~27 h and the times were positively correlated with stress duration *w* (Figure 4D). Viability decreased with increasing stress duration *w* for pH 5 and pH 10 stress pulses. With pH 4 and 11 stress pulses, viabilities decreased rapidly with stress duration *w*. After 30 min of stress, no viable cells were found (Figures 4A,C). After 5 min at pH 4, the viability was close to ~100%. After a 10 min stress pulse, the viability decreased to only ~20%; with a 10 min stress pulse at pH 11, the viability decreased to only ~70%. Figure 4 shows two main results concerning the viabilities of the colonies. First, the viability decreased between 2 and 6 h after stress duration *w* with pH 10 (Figure 4D); at longer stress durations *w*, viability remained constant at ~20%. Second, a rapid decrease in viability to ~20% was observed for pH 4 stresses lasting between 0 and 5 min (Figure 4A). Afterward, the viability decreased continuously to 0% after 30 min of stress.

### Single-Cell Growth Behavior and Adaptation During the First Post-stress Division

After investigating the time required for the first post-stress division, the regrowth behaviors of single cells before the first post-stress divisions were studied in more detail, i.e., including cell elongation prior to division. Motivating questions were as follows: Are there different physiological adaptation states after pH stress? Do cells stay in a distinct lag phase and restart growth with pre-stress growth rates, or do cells restart with a slower growth rate?

The elongation behavior of individual cells was examined by measuring the cell length over time until the first post-stress division occurred after a single pH stress pulse. Three different physiological states regarding regrowth were found after a pH stress pulse, which can be visualized in live-cell imaging. For acidic stress, such as at pH 5 (Figures 5A,C), the first physiological state included cells that started elongation immediately after stress durations *w* of 2 h (Supplementary Video S1) and 6 h (Supplementary Video S2). In this physiological state, two subpopulations were found: first, cells with exponential elongation (Figures 5E,G—cell number A.2) and second, cells with linear/slow elongation (Figures 5A,E,G—cell number A.3). Cells in the second physiological state showed a lag phase for regrowth that ranged from min to several h before cell elongation started (Figures 5A,E,G—cell number A.1). After the lag phase, the cells regained the maximum growth rate, indicating that full cell integrity was obtained after a short adaption phase. Cells in the third physiological state no longer elongated after a 2 h stress pulse of pH 5 (see Supplementary Table S1—cells not shown in Figure 5). After a stress duration *w* of 9 h (Figure 5E), two physiological states (physiological states 2 and 3) were observed. The cells showed broadly distributed lag phases. The physiological state distribution for pH 5 stress pulses showed the three observed physiological states (Supplementary Figure S6). After 2 h of a pH 5 stress pulse, ~90% of the cells belonged to the first and second physiological states, and only a small portion of the cells were in the third physiological state. With increasing stress duration *w*, the distribution shifted to physiological states 2 and 3; however, after a 9 h stress pulse, only ~ 5% of the cells belonged to the second physiological state, and most of the cells belonged to physiological state 3 (Supplementary Figure S6—pH 5). The live/dead staining confirms these results. After 2 h stress only small fractions of cells are stained (see Supplementary Figure S8A), with increasing stress duration *w*, 6 h and 9 h, the fraction increases (Supplementary Figures S8C,E). After a 2 h stress pulse, ~2% of the cells showed a fluorescence signal, indicating dead cells (Supplementary Figure S8A). In addition, it was found that a total of ~17% of the cells did not divide after the stress pulse. For the 6 h pH 5 stress pulse, we found similar results, ~30% of the cells were stained (Supplementary Figure S8C), but still ~86% of the cells showed no growth after the stress and are in the third physiological state. After the 9 h stress pulse of pH 5, ~19% of the cells were stained (Supplementary Figure S8E) and a total of ~90% of the cells showed no division after the stress pulse. At pH 10, only the second and third physiological states were found (Figures 5B,D): first, cells showed lag phases before elongation (2–14 h lag time) (Figure 5F). These cells exhibited an extended lag phase of up to 14 h until they began to elongate, and they divided at ~19 h after pH stress. Other cells started their elongations after a short growth lag phase (Figures 5D,F—cell number B.1). This physiological state can be divided into two subpopulations: first, cells that started an exponential elongation directly after the lag phase (Figures 5D,F,H—cell number B.2 and Supplementary Figure S5), and second, cells that started regrowth with linear elongation after the lag phase (Figures 5F,H—cell number B.3). Regardless of the physiological growth state, except for the first post-stress division, all daughter cells were viable and had division times of 69.7 ± 6.3 min (see Supplementary Figure S7). Approximately 99% of the single cells belonged to the second physiological state after a 2 h stress pulse (Supplementary Figure S6—pH 10 and Supplementary Video S3). Cells belonging to the third physiological state did not divide after stress. After a 6 h stress pulse at pH 10 (Supplementary Video S4), 90 cells failed to survive (see Supplementary Table S1—non-dividing cells not shown in Figure 5). These results can be confirmed with the live/dead staining. After 2 h of stress, no stained cells are observed (Supplementary Figure S8B); as the duration of stress increases (6 h), the proportion of stained cells increases (Supplementary Figure S8D). The 2 h stress pulse with pH 10 showed no staining of the cells (Supplementary Figure S8B), but ~6% of the cells are in the third physiological state and show no growth and no staining after 48 h of cultivation, while ~94% of the cells continue to grow after the stress. During the 6 h stress pulse with pH 10, ~7% of the cells were stained (Supplementary Figure S8D), confirming the death of the cells. Another ~60% of the cells do not continue to grow after the stress but are also not stained.

## Discussion

### *Corynebacterium glutamicum* Growth in Constant pH Environments

The results obtained with dMSCC of *C. glutamicum* growth at constant pH confirm those from former experiments performed on a bulk scale (Jakob et al., 2007; Follmann et al., 2009b). Jakob et al. (2007) found a comparable limiting pH range of 5.5–9.5 in a turbidostat fermenter using lactic acid and NaOH for pH control. Follmann et al. (2009a) reported a slightly broader pH range of 4–10. This difference may be due to the batch cultivation conditions of their study compared to the experiments performed under continuous conditions in this study. Batch cultivations are typically characterized by changing conditions, e.g., the limiting substrate is consumed, and products and byproducts accumulate. It is possible that *C. glutamicum* exported ions and metabolites that promoted growth under pH stress during batch cultivation (Guo et al., 2019). These effects were minimal in the setup used here. Consequently, the maximum growth rate of *C. glutamicum* reported here exceeded those observed for shake flasks or bioreactors. The maximum growth rate of μ_max_ = 0.59 ± 0.01 h^−1^ at pH values of 7–7.4 (Figure 2C) is comparable to that measured with a similar setup, namely, in a perfusion-based microfluidic single-cell cultivation system (Grünberger et al., 2013).

The finding that pH adjustments with different bases had a significant influence on the growth rate, i.e., faster growth was observed when the pH was adjusted with KOH or H_3_PO_4_, may be explained by the fact that pH control altered ion concentrations in the medium, which may have affected cells due to different transmembrane ion gradients. With concentrations between 200 and 800 mM, K^+^ is the most abundant ion in the cytoplasm of *C. glutamicum*. K^+^ ions have been shown to be important for acidic stress tolerance (Follmann et al., 2009a), whereas under alkaline conditions, a higher K^+^ concentration had no effect on growth (Follmann et al., 2009a). The *C. glutamicum* mineral salt medium had a very low concentration of Na^+^ ions because they have a negative effect on growth. The Na^+^/H^+^ antiporter Mrp1 maintains a low intracellular Na^+^ ion concentration. Deletion of its gene or chromosomal replacement of lysine 299 in the Mrp1A subunit increased the intracellular Na^+^ ion concentration and led to a more alkaline intracellular pH value, which strongly attenuated growth (Xu et al., 2018). The finding that pH adjustments with different acidic conditions had a significant influence on the growth rate, i.e., a lower growth was observed when the pH was adjusted with HCl rather than with H_3_PO_4_, may be explained by the fact that the salt concentration in the medium increased. Here, the internal osmolality was presumably increased by the accumulation of organic solutes to counteract dehydration (Eggeling and Bott, 2015). In addition, gene expression adapts to hyperosmotic conditions (Wood, 1999).

### *Corynebacterium glutamicum* Growth in Single pH Stress Pulse Experiments

Detailed analyses of survival after pH stress pulses do not appear in the literature for *C. glutamicum*, but *E. coli* and *B. subtilis* have been studied in some detail (Slonczewski et al., 2009; Wilks et al., 2009; Martinez et al., 2012). Appropriate tools required to perform these analyses at the single-cell level have been missing so far. The few reported *E. coli* studies are difficult to compare with this study because they did not follow cell elongation and division but monitored how the cytoplasmic pH changed and recovered after changes in the extracellular pH. Moreover, the applied stresses involved only small pH shifts of 0.5–2.0 units. Martinez et al. (2012) observed the cytoplasmic pH after an acidic pH shift using ratiometric pHluorin, and they showed that pH homeostasis varied among the studied bacteria. An external pH shift from pH 7.5–6 led to recovery of *E. coli* cytoplasmic pH within 7 min after the shift, with only 3% failing to recover. The cytoplasmic pH of *E. coli* decreased by 1.5 pH units as a consequence of shifting the external pH from 7.5 to 5.5. The cytoplasmic pH started to recover after 10 sec; however, this process was biphasic and showed a rapid recovery for 0.5 min followed by a gradual recovery for 4 min, until a pH of 7.4 was reached (Zilberstein et al., 1984; Slonczewski et al., 2009). Wilks et al. (2009) analyzed the growth of *B. subtilis* after pH shifts and found that after a pH shift from 8.5 to 6, cells started to grow rapidly again after a short lag phase. However, after a pH shift from 6 to 8.5 *B. subtilis* showed a longer lag phase. Similar to the results obtained with *B. subtilis*, our study also showed long lag phases lasting several hours after alkaline stress, and the lag times increased with increasing stress duration *w*.

For all pH stress pulses, lag phases did not only increase with increasing stress pulse duration, but a broadening of the distribution for first post-stress division times was also found, which may indicate increasing heterogeneity among genetically identical cells coping with pH stress. At pH 4 and 11, a strong effect was observed after pH stress, as evidenced by the rapid decrease in viability. After 30 min at pH 11, no cells survived. One possible mechanism could involve mycothiol, which protects cysteine and methionine residues of proteins from ROS by reversible S-mycothiolations. This allows for fast protein regeneration after acidic stress (Liu et al., 2016). In alkaline environments, no ROS protection is necessary (Follmann et al., 2009b).

The reductions in the viabilities at pH 4 and 11 could be similar to the thermal death curves observed for various bacterial species. The viability at pH 4 decreased at a rate similar to that found in the thermal death curves for *Cl. botulinum*, for which a rapid decrease (<2 min) in cell survival was observed at 121°C (Peleg et al., 2005). For *B. sporothermoduranes*, a slower decrease was found for the 121°C survival curve, similar to the decrease in viability observed here at pH 11. For *C. glutamicum*, no studies on thermal death points could be found. pH values of 4 and 11 could thus have a disinfecting effect within a culture.

In the last part of the study, cell elongation prior to the first post-stress division was investigated in more detail. Three different physiological states were found after a pH 5 stress pulse. It is possible that the preculture already contained subpopulations that differed in their preadaptations to pH stress. Alternatively, the three physiological states may have arisen stochastically. In the first subpopulation (Figure 5C-1 and 2), cells did not seem to be significantly affected by the stress pulse. These cells may have been preadapted to environmental changes so that no damage was induced by the pH stress (Figure 5C—blue cells). The cells in the second physiological state might not have been adapted to the environmental changes and showed a lag phase. In this case, proteins may have been denatured, PMF was compromised, and further damage from pH stress may have occurred, requiring time to repair. The third physiological state is that of non-growing cells. Experiments involving staining of dead cells (Supplementary Figure S8) showed that some cells were stained and no longer divided. Here, the cell membrane was no longer intact because the substance has penetrated the cells. Cells were also discovered that were not stained and no longer divided after the stress pulse. These cells were also included in the non-growing physiological state. In this work, it was not possible to say whether the cells were dead or dormant. It is possible that the staining did not work properly or that the cell membrane was intact even though the cells were dead, meaning that the cells could not be stained. It is also possible that the cells were in a dormant state. After 48 h of cultivation, no cell division was observed. Cells with this physiological status may have impaired PMF and further damage due to pH stress that was not repairable (Figure 5C-3). *C. glutamicum* has two open reading frames, *rpf1* and *rpf2*, which code for proteins similar to the essential resuscitation promoting factor (Rpf) from *M. luteus*, which would enable renewed growth through the addition of additional substances (Mukamolova et al., 1998; Hartmann et al., 2004). At pH 10, phenotypic heterogeneity was evident because of the different lag phases. The live/dead staining shows for the third physiological state also a combination of impaired (probably dead) and dormant cells. Here are around 60% of the cells which are not strained after a 6 h stress pulse and do not divide after the stress. There are two explanations for this: False negative due to an error in staining (Rosenberg et al., 2019) or delayed regrowth that exceeds the cultivation time. Physiological states similar to acidic stress have been observed, with one difference: all physiological states had a growth lag phase (Figure 5D-1–3). This observation of different physiological states can be compared with the physiological states observed after antibiotic stress of *E. coli* (Kim et al., 2018). There, five different physiological states were found. Three of these results were similar to those we observed, so similar physiological states were observed with *C. glutamicum*.

Especially under stress conditions, subpopulations with genotypic or phenotypic adaptation mechanisms are beneficial for the survival of a population (Ryall et al., 2012; Bremer and Krämer, 2019). At alkaline pHs, compared to acidic stress, there was a growth lag phase (no direct regrowth observed) during which the cells did not grow and stagnated for several hours before length growth was observed. Here, it is possible that a genetic response to pH stress is necessary, which requires time. For example, in the case of osmotic stress (Wood et al., 2001), a fast response involving transport and uptake of compatible substances from the medium is first carried out, and then a genetic response is triggered. Acidic stress may require biochemical reaction mechanisms (e.g., mycothiols, protein refolding, iron transport, etc.) with which to respond to the stress pulse. In *B. subtilis*, more genes were upregulated by alkaline stress than by acid stress, which requires survival of the pH value. In this case, little or no growth lag was observed after acid shift, which is comparable with the results shown here for *C. glutamicum* (Wilks et al., 2009).

The observed physiological states need to be examined further in future studies, e.g., with a fluorescent signal coupled to PMF (Novo et al., 1999), so that loss of capability or damage occurring during or after pH stress can be analyzed in more detail. Furthermore, cytoplasmic pH values can be determined with pH-sensitive fluorescent proteins (Haynes et al., 2019). Further insights into heterogeneity and adaptive behaviors in response to pH stress pulses could be gained from fluorescence-based sensors that reproduce the cellular pH value (Miesenböck et al., 1998; Mahon, 2011). Non-invasive detection of cytoplasmic pH could answer questions about how long *C. glutamicum* is able to maintain homeostasis and how physiology is affected after pH stress pulses when pH homoeostasis collapses.

The microfluidic dMSCC technology used in this study offers numerous advantages for analyses of cellular behaviors. These include defined environments, which can be studied precisely down to a pulse/oscillation of 5 sec, as well as analyses of single-cell dynamics. Further and detailed insights into heterogeneity in single-cell adaptations occurring after pH stress pulses could be obtained by using single-cell growth channels, which are often referred to as mother machines (Wang et al., 2010), instead of the cultivation chamber used in this work. In mother machines, single cells can be grown over several generations, and detailed dynamic single-cell studies can be performed. This allows quantification of single-cell growth (i.e., elongation and time until cell division) over many generations and allows determination of whether and to what extent the pH stress pulse affects, for example, the maximum growth rates in the generations following the stress pulse. In the future, these systems could even be used to investigate the behavior of single cells reacting to an oscillating pH stress or to multiple (two or three pulses) pH stresses. Here, questions such as how individual cells respond to oscillating pH stress can be answered.

## Conclusion

This study investigates the influence of different pH pulses at the single-cell level. dMSCC was used to analyze the growth response of *C. glutamicum* at the single-cell level to different single stress pH values. This study confirms the results from bulk-scale experiments performed at constant pH. Long-term cultivations at non-optimal pH values ranging between 5 and 10 were performed to analyze the growth of *C. glutamicum*. Colony growth was observed in the pH range 6–8, which is consistent with lab-scale systems. New insights were developed into the growth behaviors occurring with non-optimal pHs and after different pH stress pulses, which had not been possible before. Different single stress pulses with pH values between 4 and 11 were applied to analyze how long cells could survive at these pH values and the heterogeneity with which cells continued to grow after the stress pulses. *Corynebacterium glutamicum* survived for up to 9 h at pH 5, and the viability decreased continuously after 5 h of stress to ~6% after 9 h of stress. At pH 10, the viability was constant at ~20% after 6 h of stress. It has been shown that the growth lag phase resulting after acidic stress was shorter than that after alkaline stress. In addition, the growth behavior of single cells after stress duration *w* was analyzed. It was found that the time of the first post-stress division varied widely at all pH values, indicating heterogeneous behavior. Three physiological states were observed for acidic pH values: in the first, cells were preadapted to the stress pulse lasting for a duration of 6 h, and length growth could be exponential or linear. Second, cells showed a lag phase before cell elongation and division began. Third, cells did not divide after the stress pulse. At alkaline pH values, only two physiological states were found: first, cells stagnated for several hours until (exponential or linear) elongation started. Second, cells did not divide after the stress pulse. This study provided the basis for microbial physiological states and adaptation to pH stress pulses and laid a foundation for further studies under fluctuating and oscillating pH conditions.

## Data Availability Statement

The raw data supporting the conclusions of this article will be made available by the authors, without undue reservation.

## Author Contributions

ST and AG contributed to the conceptualization and design of the study and wrote the first draft. ST designed the methodology. LB and ST performed the experiments and analyzed the data. ST, LB, VW, and AG interpreted the results, edited, and reviewed the manuscript. AG provided the financial resources. All authors contributed to the article and approved the submitted version.

## Funding

We acknowledge the financial support of the German Research Foundation (DFG) and the Open Access Publication Fund of Bielefeld University for the article processing charge. The funding bodies had no role in the design of the study or the collection, analysis, or interpretation of data or in writing the manuscript.

## Conflict of Interest

The authors declare that the research was conducted in the absence of any commercial or financial relationships that could be construed as a potential conflict of interest.

## Publisher's Note

All claims expressed in this article are solely those of the authors and do not necessarily represent those of their affiliated organizations, or those of the publisher, the editors and the reviewers. Any product that may be evaluated in this article, or claim that may be made by its manufacturer, is not guaranteed or endorsed by the publisher.
